# *Rhinella marina* oocytes: a suitable alternative expression system for functional characterization of aquaglyceroporins

**DOI:** 10.1038/s41598-018-37069-6

**Published:** 2019-01-10

**Authors:** Vania Rojas, Yulexi Y. Ortiz, Sheridan Rodríguez, Vladimir Araque, Alexis Rodríguez-Acosta, Katherine Figarella, Néstor L. Uzcátegui

**Affiliations:** 1 Laboratorio de Inmunoquímica y Ultraestructura, Instituto Anatómico “José Izquierdo”, Universidad Central de Venezuela, Caracas, Venezuela; 2 Institute of Physiology, Department of Neurophysiology, Eberhard Karls University of Tübingen, Tübingen, Germany

## Abstract

Amphibian oocytes have been extensively used for heterologous expression of membrane proteins for studying their biochemical and biophysical properties. So far, *Xenopus laevis* is the main amphibian used as oocytes source to express aquaglyceroporins in order to assess water and solutes permeability. However, this well-established amphibian model represents a threat to the biodiversity in many countries, especially in those from tropical regions. For that reason, the import of *Xenopus laevis* is subjected to strict control, which essentially has restricted its use in these regions. Therefore, a wider variety of expression systems for aquaglyceroporins is needed. *Rhinella marina* is extensively distributed in the Americas and its native range spreads from South America to Texas, US. Here we report the use of *Rhinella marina* oocytes as an alternative expression system for aquaglyceroporins and demonstrated its suitability to determine the permeability to water and non-ionic solutes. *Rhinella marina* oocytes were able to functionally express channels from human and the protozoan pathogen *Trypanosoma brucei*, two very distant organisms on the evolutionary scale. Permeability values obtained from *Rhinella marina* oocytes expressing members of aquaporin family were similar and comparable to those values reported in the literature for the same channels expressed in *Xenopus laevis* oocytes.

## Introduction

The aquaporin protein family are pores located in biological membranes, which allow the passage of water (orthodox aquaporins) and other small uncharged solutes such as glycerol (aquaglyceroporins)^1^. The importance of aquaporins can be inferred from their wide distribution throughout nature from bacteria to human. Interestingly, they are highly abundant in mammals and plants, 13 different and more than 30 aquaporins, respectively^2^. These proteins have innumerable functions, they have even also been shown to be involved in the passage of drugs through membranes^3^.

Heterologous expression of orthodox aquaporins and aquaglyceroporins in *Xenopus laevis* (*X. laevis*) oocytes is one of the most widespread systems used for the study of these proteins. In 1971 it was demonstrated for the first time that *X. laevis* oocytes were able to translate foreign mRNA^4^, since then, they have become a popular tool to study membrane proteins from different origins, ranging from virus and bacteria to plants and animals^5–10^. The appearance of a method for the determination of water permeability in *X. laevis* oocytes had to wait until the beginning of the nineties. Fischbarg´s and Verkman´s groups developed assays based on the measurement of the swelling rate of the oocyte in response to osmotic gradients^11,12^. In 1992, the Agre´s group, applying a similar methodology, was able to demonstrate for the first time, the molecular identity of a water channel, the human aquaporin 1 (hAQP1)^13^. From that time on, this method has been useful and extensively used in the characterization of proteins belonging to the aquaporin family and, transporters associated with water movement through membranes^14^.

The *X. laevis* oocytes system is a convenient and powerful tool that has been used for the analysis of a wide range of molecular features in proteins; structure/function, mutations, regulation, protein-protein interactions, among others^15–18^. However, the use of a unique system for functional expression of aquaglyceroporins may face a number of constraints. Indeed, low or not protein expression, endogenous protein background, protein interactions, among others have been reported in *X. laevis* oocytes^5,19^. Additionally, there are several disadvantages of the *X. laevis* system that must not be underestimated, for instance, restriction to import this amphibian in some countries, susceptibility to sicknesses, maintenance costs, the impact of housing and husbandry conditions as well as seasonal variability on the oocytes quality^20^. Therefore, it is reasonable to establish others amphibian oocytes as alternative systems for functional characterization of membrane proteins. This will allow, according to the respective protein’s features and laboratory conditions, to choose the most appropriate experimental model.

*Rhinella* (*R.) marina* (formerly *Bufo marinus*), the so-called cane toad, is for many reasons one of the most suitable amphibian candidates to substitute *X*. *laevis* as an alternative source of oocytes to analyse water and uncharged solutes permeability. This toad has a very high rate of reproduction, a female can spawn more than 30,000 eggs in a single clutch^21^. This amphibian is widely distributed and its native range extends from South and Central America to Texas (USA). This geographical distribution makes *R. marina* particularly attractive as an amphibian model for tropical countries, where *X. laevis* represents a clear threat to the biodiversity of the region and, therefore, its import is highly restricted. Additionally, *R. marina* possesses a robust capacity of adapting to new environments, which was demonstrated in several occasions by its introduction to different geographical regions around the world, e.g. Florida, Caribbean islands, Hawaii, and Australia^22^. Importantly, it has been already demonstrated that its oocytes can translate foreign mRNA from different species^23–25^. However, *R. marina* oocytes have been neither validated as an expression system for aquaglyceroporins nor analyzed their usefulness for determination of water and solutes permeability. In this study, we evaluated whether *R. marina* oocytes can express orthodox aquaporins and aquaglyceroporins from different species distant on the evolutionary scale and if the biological properties of *R. marina* oocytes are suitable for standard osmotic swelling assays in order to determine water and solutes permeability.

## Results and Discussion

Protocols to obtain oocytes from *R. marina*, the defolliculation technique used, their maintenance, and injection were optimized (see material and methods). In general, the procedure was similar to those reported in the literature for *X. laevis, X. boriales, and R. marina*^24–28^. *R. marina* oocytes were injected with cRNA encoding either a human orthodox aquaporin (hAQP1) or *T. brucei* aquaglyceroporins (TbAQPs), in order to evaluate whether these cells are able to express aquaglyceroporins and are appropriate to perform water and non-charged solutes permeability measurements. Control oocytes were injected with water.

### Validation of *R. marina* oocytes as a model for aquaglyceroporin water permeability determination

**Figure 1:**
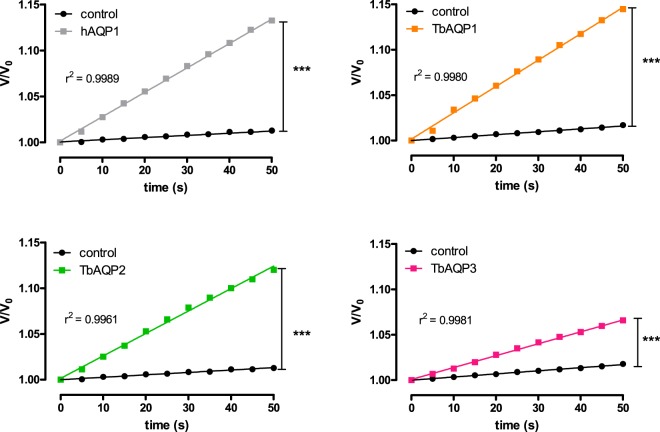
Evaluation of the linearity of the standard oocytes hypo-osmotic swelling assay in *R. marina* oocytes. Oocytes of *R. marina* injected with RNA encoding for hAQP1, TbAQP1, 2 or 3 were subjected the standard oocytes hypo-osmotic swelling assay and the relative volume change (d(V/V0)/dt) was monitored. Control cells were injected with water.

For evaluation of water permeability, R. *marina* oocytes expressing hAQP1 or TbAQPs were subjected to a hypo-osmotic swelling assay under a 140 mOsm osmotic gradient (i.e. a fast transfer of the oocytes from 200 to 60 mOsm of ND96 solutions). Under these conditions, an immediate increase of water permeability was observed for all aquaglyceroporins assayed. At the beginning of the osmotic challenge, the oocyte swelling in the time produced a straight-line with linear regression R^2^ values near to 1 (Fig. 1). Control oocytes showed a negligible swelling when subjected to the same condition (Fig. 1). These results are similar to those published for *X. laevis* oocytes injected with either total RNA or cRNA from different aquaglyceroporins^12,13,29^. Our findings validate the suitability of *R. marina* oocytes as an orthodox aquaporin and aquaglyceroporins expression system for water permeability determination.

### Parameter optimization for the water permeability assay in *R. marina* oocytes

For further characterization of the *R. marina* oocytes as a system for water permeability determination, hAQP1 and TbAQPs were expressed in these cells and parameters as protein expression kinetic, amount of injected hAQP1-cRNA, and values reproducibility were evaluated and optimized:

### Optimization of parameters for hAQP1

**Figure 2:**
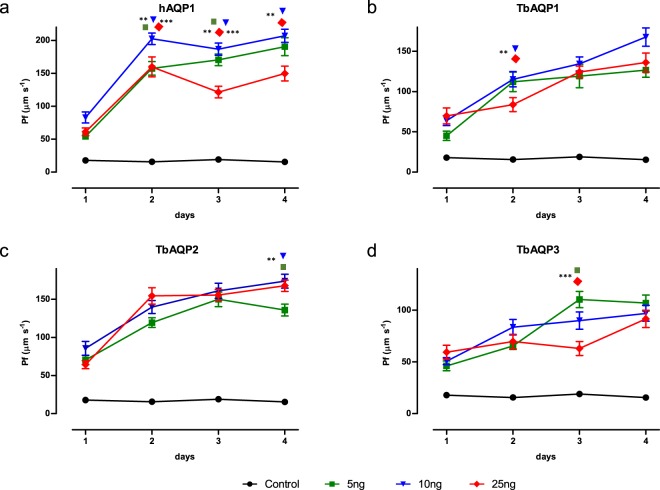
Water permeability values in *R. marina* oocytes expressing hAQP1, TbAQP1, 2, or 3. *R. marina* oocytes were injected with water (control cells) or 5, 10, and 25 ng of cRNA encoding for hAQP1, TbAQP1, 2, or 3 and subjected to the standard oocytes hypo-osmotic swelling assay every day. Water permeability values were obtained according to Pf equation. Results are shown as mean values ± standard error (SE). Kruskal-Wallis statistical test was used and the statistical significance is represented as >0.05 (*), 0.01 (**), and 0.001 (***). Pf values of oocytes expressing hAQP or TbAQPs compared to control oocytes were in all cases statistically different. For simplicity, the figure only shows the statistical significance of the cRNA amounts compared among them starting from the second day. For instance, (**a**) day 2 (green square, **, inverted blue triangle, ***, and orange diamond) indicates that 10 ng is statically different from 5 ng (**) and from 25 ng (***), but there are no differences between 5 ng and 25 ng.

As shown in Fig. 2, control oocytes evaluated between days 1 to 4 post injection (p.i.) showed a negligible osmotic swelling. The values obtained over these days were constant and compatible with simple membrane diffusion (between 15.4 and 17.9 μm.s^−1^). Interestingly, these values are comparable to those reported in the literature for *X. laevis* oocytes^13,17^. Expression of hAQP1 led to a very fast cell swelling as compared to control oocytes (Fig. 2a). *R. marina* oocytes injected with 5 ng, 10 ng or 25 ng of hAQP1-cRNA showed a statistically significant increase in membrane permeability when compared to control oocytes. Interestingly, the lowest permeability was always found on the first day p.i. This may be explained by the large size of these oocytes, which implicates that it takes some time for the translation machinery to produce and localize the new protein into the plasma membrane. Using 10 ng or 25 ng hAQP1-cRNA, the maximum hAQP1 expression was reached on the second day p.i. and maintained until the fourth day, while 5 ng needed 3 days for the maximal protein expression. Similar results have been reported for hAQP1 and other aquaporins expressed in *X. laevis* oocytes^12,13,29,30^. The highest permeability value was obtained at day 4 when 10 ng of hAQP1-cRNA were injected. Under these conditions, the increment of oocytes swelling was 13.4-fold higher than that of control oocytes and the Pf value calculated for hAQP1 was 207 ± 59 μm.s^−1^, which is similar to that reported in the literature for hAQP1 in *X. laevis* oocytes (210 ± 41 μm.s^−1^) (Table 1)^13^.

**Table 1:** Comparison between water permeability values of hAQP1 and TbAQPs obtained in *X. laevis* and *R. marina* oocytes.

	Pf *(*μm.s^−1^) in *R. marina*	Pf *(*μm.s^−1^) in *X. laevis*	Difference (%)	References (*X. laevis)*
hAQP1	207	210	1.4	Preston *et al*. 1992
TbAQP1	168	172	2.3	Uzcátegui *et al*. 2004
TbAQP2	178	139	22	Uzcátegui *et al*. 2004
TbAQP3	110	164	33	Uzcátegui *et al*. 2004

### Optimization of parameters for TbAQP1, 2, and 3

For further validation, aquaglyceroporins from the protozoan pathogen *T. brucei* were expressed in *R. marina* oocytes and the standard hypo-osmotic swelling assay was performed and parameters were optimized (Fig. 2b–d). All three TbAQPs generated a high rate of oocyte swelling, showing a volume increase between 6.9- and 11.3-fold with respect to control cells. For TbAQP 1 and 2, the kinetics of protein expression was similar to that of hAQP1, i.e. the lowest expression was obtained on day 1 and the optimal expression was reached on day 2 and stable thereafter (Fig. 2b,c). TbAQP3 showed the same trend, however, slightly more variable, where the day 4 was the best for the three amounts of TbAQP3-cRNA assayed (Fig. 2d).

In some cases, the differences between the evaluated parameters were statistically non-significant. However, a trend is observed that is also valid for hAQP1. Taking together, 10 ng of injected cRNA produced the best results for all aquaglyceroporins, i.e. the highest permeability value were obtained using 10 ng of TbAQP1-cRNA, 10 or 25 ng of TbAQP2-cRNA, and 5 or 10 ng of TbAQP3-cRNA. The optimal days for experiments were day 3 and day 4 p.i., and the highest permeability values from all recombinant TbAQPs were: 168 ± 49 μm.s^−1^, 178 ± 54 μm.s^−1^ and 110 ± 46 μm.s^−1^ (mean ± standard deviation) for TbAQP 1, 2 and 3, respectively (Fig. 2b–d)(Table 1). The Pf value of TbAQP1 in *R. marina* oocytes is similar to the one obtained for *X. laevis* oocytes, 172 μm.s^−1 ^^26,31^. Results for TbAQP2 and 3 are comparable with data reported in the literature, being 22% higher or 33% lower than their counterparts, respectively^26,31^. These differences are in the same range or even lower than those observed for some aquaporins expressed in *X. laevis* oocytes in different laboratories. Although the results in each laboratory (intra-laboratory) are reproducible, experimental conditions among different laboratories (inter-laboratory) may generate a high variability in Pf values, e.g., the Pf of rAQP3 (aquaglyceroporin 3 from rat) obtained in *X. laevis* oocytes ranges between 218 μm.s^−1^ and 55 μm.s^−1^, which corresponds to a variability close to 75%^30,32–34^. Similar examples can be found in the literature for others AQPs^13,35,36^.

The good agreement of water permeability data from hAQP1 and TbAQPs expressed in *R. marina* oocytes with what has been published in the literature for the same aquaporins expressed in *X. laevis* oocytes, validate this system as an alternative and easy approach for water permeability determination of aquaglyceroporins. Furthermore, it may be useful for others membrane proteins that, in addition to their role, can work as water channels as well (e.g carriers for glucose)^37^.

### Validation of *R. marina* oocytes as a model for aquaglyceroporin solute permeability measurement

Glycerol was chosen as substrate and tested with a standard iso-osmotic oocyte swelling assay (see materials and methods), as proof of concept for non-ionic solute permeability measurements in *R. marina* oocytes. The experiments were performed according to the parameters already optimised for water permeability: i.e. injection of 10 ng of cRNA and measurements between days 2–4 p.i. The standard iso-osmotic oocyte swelling assay for solute permeability measurements in *X. laevis* oocytes is affected by the water transport capacity of the assayed aquaglyceroporin^16,26,30^. To evaluate if a similar phenomenon can also occur in *R. marina* oocytes, TbAQP2 and TbAQP3 were chosen to be tested for glycerol permeability, as examples of aquaglyceroporins with high and middle permeability for water (Figs 1 and 2).

**Figure 3:**
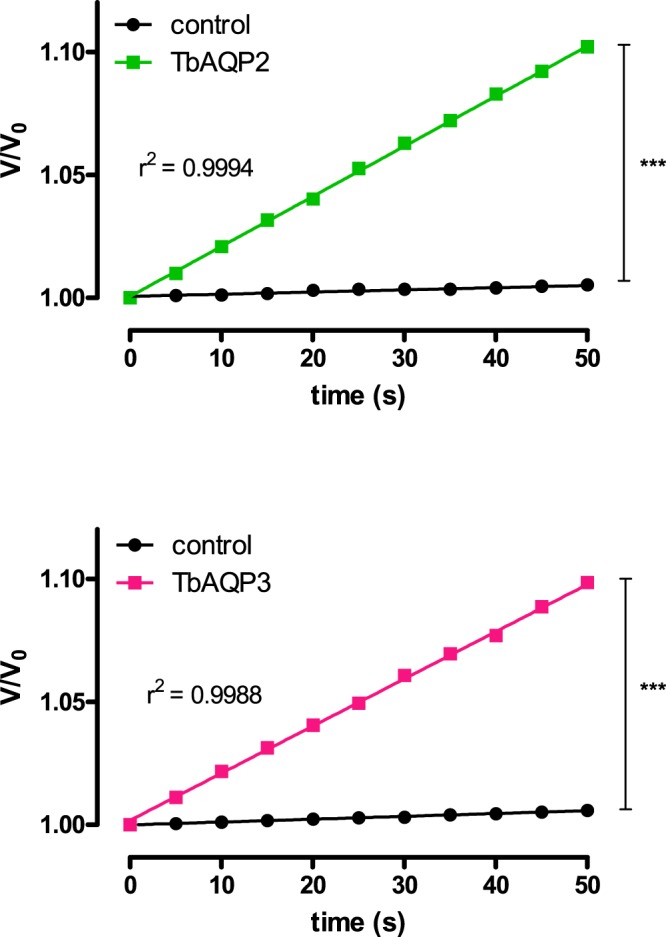
Evaluation of the linearity of the standard oocytes iso-osmotic swelling assay in *R. marina* oocytes. Oocytes of *R. marina* expressing TbAQP2 or 3 were subjected to the standard oocytes iso-osmotic swelling assay and the relative volume change (d(V/V0)/dt) was monitored. Control cells were injected with water.

*R. marina* oocytes expressing TbAQP2 or TbAQP3 were transferred quickly from the ND96 into ND96^gly+^, an iso-osmotic solution in which 65 mM NaCl was substituted by 130 mM of glycerol (see material and methods). Under these conditions, a glycerol gradient is created maintaining the iso-osmolarity. The influx of glycerol across the aquaglyceroporin into the oocytes changes the inside osmolarity of the cells and produces a concomitant water entry, leading to the oocyte’s swelling proportionally to its glycerol permeability^16,17,26,30,38^. As shown in Fig. 3, when oocytes injected with 10 ng of TbAQP2-cRNA or TbAQP3-cRNA were subjected to the referred glycerol gradient, the association between swelling versus time displayed a linear behaviour with R^2^ values close to 1 (Fig. 3), similar as it occurred for water permeability measurements by hypo-osmotic swelling assay (Fig. 1).

**Figure 4:**
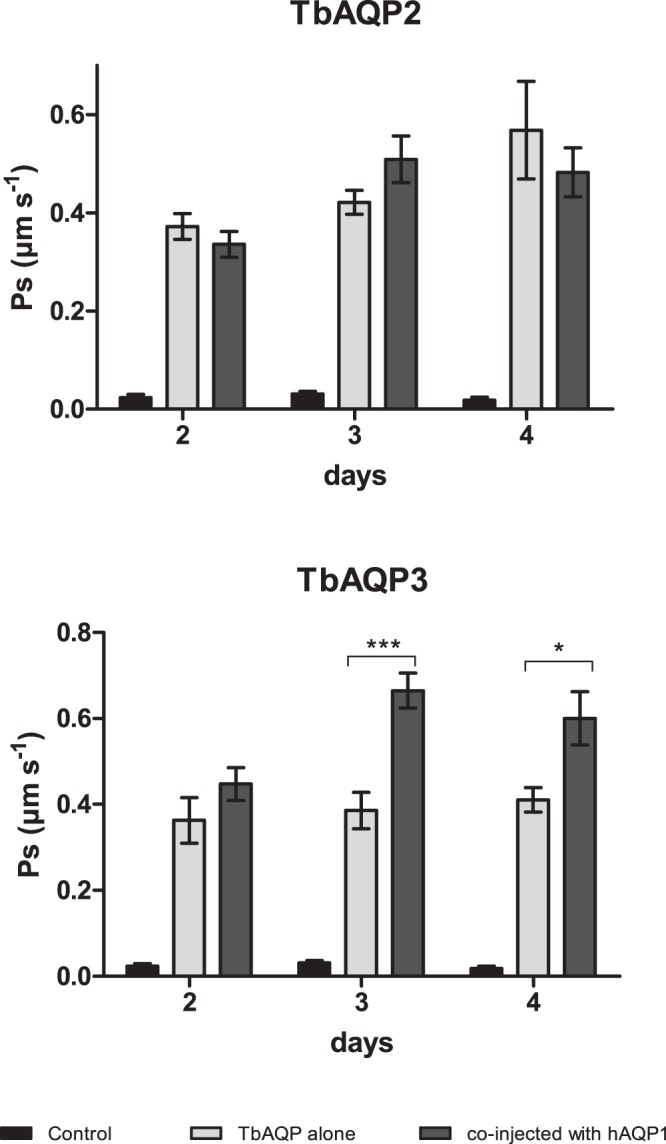
Glycerol permeability values in *R. marina* oocytes expressing TbAQP2 or 3. *R. marina* oocytes were injected with water (control cells), 10 ng of TbAQP2-cRNA, or 3-cRNA (alone or co-injected with 5 ng of hAQP1-cRNA). At day 2, 3, and 4 p.i., oocytes were subjected to the standard iso-osmotic swelling assay. Glycerol permeability values were obtained according to Ps equation. Results are shown as mean values ± standard error (SE). The t-student statistical test was used and the significance was represented with asterisks as follows, p < 0.05 (*), 0.01(**) and 0.001(***).

The permeability for water may play a role in the iso-osmotic oocyte swelling assay, particularly when conductance for water of the aquaglyceroporin to be evaluated is limited. It produces a partial oocyte swelling, showing biased results towards lower values when subjected to substrate gradient^26,30,38^. To investigate this fact in the *R. marina* oocytes system, oocytes from this amphibian were injected with water (control) or with TbAQPs-cRNA alone or in combination with hAQP1-cRNA and evaluated by standard iso-osmotic oocyte swelling assay. Since optimal conditions for expression were previously determined by the water permeability experiments, only 10 ng of TbAQP2- or 3-cRNA, and 5 ng of hAQP1-cRNA were co-injected and kinetic of permeability studies were performed at 2–4 days p.i. As TbAQP1 and 2 showed similar high-water permeability values (Fig. 2b,c), only TbAQP2 was chosen for expression. TbAQP3 was also evaluated as it showed the lowest permeability of the three TbAQPs (Figs 1 and 2). Figure 4 summarizes the results obtained. Control oocytes exhibited a minimal swelling displaying a very low Ps, between 0.02–0.03 μm.s^−1^, consistent with simple membrane diffusion. *R. marina* oocytes injected with TbAQP2- or TbAQP3-cRNA exhibited a high permeability for glycerol, statistically different than that observed for control cells. Results of oocytes expressing TbAQP2 alone or together with hAQP1 were not statistically different, meaning that the intrinsic water permeability of this aquaglyceroporin did not limit the glycerol measurements in the standard iso-osmotic oocyte swelling assay. By contrast, *R. marina* oocytes expressing TbAQP3 alone or co-injected with hAQP1 displayed clear statistically significant differences in the permeability values. At days 3 and 4, oocytes expressing TbAQP3 alone showed consistently 42% and 32% lower permeability to glycerol than those shown by oocytes co-injected with hAQP1, respectively. Therefore, the TbAQP3 glycerol permeability measurement was partially limited due to TbAQP3 middle water permeability. The highest glycerol permeability values for TbAQP2 and 3 were 0.57 μm.s^−1^ (day 4) and 0.67 μm.s^−1^ (day 3), respectively. TbAQP2 and 3 displayed 47% and 29% lower permeability than those reported in the literature for the same aquaglyceroporins expressed in *X. laevis* oocytes (Table 2)^26,31^. However, as discussed above for the observed differences in water permeability, the different glycerol permeability values are still within the range of data observed for a defined aquaglyceroporin expressed in *X. laevis* oocytes in different laboratories^29,32,33^.

**Table 2:** Comparison between glycerol permeability values of TbAQPs obtained in *X. laevis* and *R. marina* oocytes.

	Ps *(*μm.s^−1^) in *R. marina*	Ps *(*μm.s^−1^) in *X. laevis*	Difference (%)	References (*X. laevis)*
TbAQP2	0.57	1.09	47	Uzcátegui *et al*. 2004
TbAQP3	0.67	0.95	29	Uzcátegui *et al*. 2004

Amphibian oocytes have been used for heterologous expression of membrane proteins to study their biophysical properties. Hitherto, apart from the popular *X. laevis*, there are other four species used as oocytes source for ion channels expression to perform electrophysiological studies, *X. borealis*, *R. marina*, *Cynops pyrrhogaster* (a Japanese fire belly newt), and *Ambystoma mexicanum* (a Mexican salamander), all of them are a reliable substitute for *X. laevis*^25,28,39,40^. However, so far, *X. laevis* is the only amphibian used as oocytes source for expressing aquaglyceroporins and study water and solutes permeability. In summary, here we report the use, for the first time, of *R. marina* oocytes as a heterologous expression system for aquaglyceroporins and validate them as a suitable alternative for water and non-ionic solute permeability determinations.

## Material and Methods

### Statement of Ethics

The procedures had approval from the authorized ethical committee of the Anatomical Institute, Central University of Venezuela (ethics approval number: FM-IA-2014/001). All experiments were carried out in accordance with the approved guidelines.

### Heterologous expression of hAQP1, TbAQP1, TbAQP2, and TbAQP3 in *R. marina* oocytes

Toad ovary removal and oocytes obtaining were performed as previously described with some modifications^16,24–28,41^. Briefly, ovarian tissue was surgically obtained from adult females of *R. marina* under anaesthesia (0.23% 3-aminobenzoic ethyl ester acid). The ovarian tissue was placed in a petri dish and dissected with scissors in small fragments. Thereafter, the oocytes were completely separated and defolliculated by treatment with 2 mg/mL collagenase type A (Roche, Germany). This procedure was monitored using a stereomicroscope. In an incubator (New Brunswick Scientific), oocytes were maintained in slight shaking at 70 rpm and 25 °C for 1 h in a calcium-free ND96 solution containing collagenase. Then a second incubation for 40–70 min was carried out with a fresh solution until defolliculation was complete. Once the connective tissue was completely detached from oocytes, collagenase was removed by washing the cells extensively with 1 L of calcium-free ND96 and then subsequent with 1 L of complete ND96. Afterwards, the biggest defolliculated oocytes were stored in the last solution at 18 °C under continuous gentle shaking, until cRNA injection (usually within the following 24 h). cRNA was then synthesized from linearized constructs (pT7TS/TbAQP1, pT7TS/TbAQP2, pT7TS/TbAQP3, and pXG-ev1/hAQP1)^13,26^ by *in vitro* transcription with the mMessage mMachine® kit (Ambion) according to the manufacturer’s instructions. The obtained cRNA was isolated by the RNeasy Mini Kit (Qiagen). For expression in *R. marina* oocytes, different amounts of cRNA from hAQP1 (5–25 ng) or TbAQPs (5–25 ng) contained in 50 nL water were injected per oocyte. For solutes permeability experiments cRNA of TbAQPs were co-injected with 5 ng of hAQP1-cRNA per oocyte (see results). Control oocytes were injected with 50 nL of water. Injected oocytes were maintained at 18 °C under continuous gentle shaking in complete ND96 until standard oocyte swelling assay was performed.

### Standard oocytes swelling assay for water and solutes permeability measurements

The standard oocytes swelling assay was performed as previously described^12,16,26,30,38,41^. The assay has two modalities: the standard oocytes hypo-osmotic swelling assay to determine water permeability (Pf) and the standard oocytes iso-osmotic swelling assay that serves to measure solute permeability (Ps). Briefly, Pf determination was performed by pre-incubation of cRNA injected oocytes in ND96 medium adjusted to 200 mOsm and then transfer to a diluted ND96 (60 mOsm). In the case of the standard oocyte iso-osmotic swelling assay for Ps determination, cRNA-injected oocytes were also equilibrated in ND96 (200 mOsm) and then transferred to ND96^gly+^ (200 mOsm), where 65 mM of NaCl was replaced by 130 mM of the glycerol in order to maintain the osmolality. Both standard swelling assays were video-monitored between 40–90 seconds (depending on swelling speed) in order to obtain the initial slope of the relative oocyte volume increase (d(V/V0)/dt). Water and solute permeability were calculated using the following equations:

$$Pf=[Vo\,x\frac{d(\frac{V}{Vo})}{dt}]/[S\,x\,Vw\,x\,(os{m}_{in}-os{m}_{out})]$$

$$Ps=[os{m}_{total}\,x\,Vo\,x\frac{d(\frac{V}{Vo})}{dt}]/[S\,x(so{l}_{out}-so{l}_{in})]$$

where, **S** represents the oocyte surface area (0.045 cm^2^), **Vo** is the initial oocyte volume (9 × 10^−4^ cm^3^), **Vw** is the molecular water volume (18 cm^3^/mol), **(osm**_**in**_ − **osm**_**out**_**)** is the osmotic gradient (outwardly directed osmotic gradient of 140 mOsm) driven by the external and internal osmolalities (osm_out_ = 60 mOsm and osm_in_ = 200 mOsm), **osm**_**total**_ is the total osmolality of the system (200 mOsm), and **(sol**_**out**_ − **sol**_**in**_**)** is the inwardly directed glycerol gradient (130 mM)^12,38^.

Pf and Ps represent the conductance of water and solutes, respectively. In this study, Pf and Ps were also used as a good estimation of the protein expression. As the measurement of the conductance of a particular aquaglyceroporin varies with its level of expression, we assumed that Pf and Ps values are an approximation to the amount of aquaporin or aquaglyceroporin functionally expressed within the plasma membrane. Although it was not the case in this study, it is important to keep in mind that, in general, expression systems have limitations and it is possible that a particular protein does not express or the measurement of its biological activity do not match its protein level.

### Statistical analysis

Statistical calculations were performed using GraphPad Prism software. Unless otherwise specified, results are shown as mean values ± standard error (SE). Student’s t-test was used when we had two sets of data following a normal distribution. In experiments using several groups, where at least one of their data set were not normally distributed, comparison among groups was performed using Kruskal-Wallis analysis. p < 0.05 was considered as statistically significant.
